# Fibroblast Activation Protein Acts as a Biomarker for Monitoring ECM Remodeling During Aortic Aneurysm via ^68^Ga‐FAPI‐04 PET Imaging

**DOI:** 10.1002/advs.202411152

**Published:** 2025-02-14

**Authors:** Chengkai Hu, Hui Tan, Yuchong Zhang, Genmao Cao, Chenye Wu, Peng Lin, Shouji Qiu, Fandi Mo, Enci Wang, Shiyi Li, Tong Yuan, Zheyun Li, Weiguo Fu, Dengfeng Cheng, Hao Lai, Xiaoyuan Chen, Lixin Wang

**Affiliations:** ^1^ Department of Vascular Surgery Zhongshan Hospital Fudan University 180 Fenglin Road Shanghai 200032 China; ^2^ Department of Vascular Surgery (Xiamen) Zhongshan hospital Fudan University Xiamen 361015 China; ^3^ Xiamen Municipal Vascular Disease Precise Diagnose & Treatment Lab Xiamen 361015 China; ^4^ Department of Nuclear Medicine Zhongshan Hospital Fudan University 180 Fenglin Road Shanghai 200032 China; ^5^ Department of Cardiac Surgery Zhongshan Hospital Fudan University 180 Fenglin Road Shanghai 200032 China; ^6^ Departments of Diagnostic Radiology Chemical and Biomolecular Engineering, Biomedical Engineering, Pharmacy and Pharmaceutical Sciences Yong Loo Lin School of Medicine and College of Design and Engineering National University of Singapore Singapore 119074 Singapore; ^7^ Clinical Imaging Research Centre Centre for Translational Medicine Yong Loo Lin School of Medicine National University of Singapore Singapore 117599 Singapore; ^8^ Nanomedicine Translational Research Program Yong Loo Lin School of Medicine National University of Singapore Singapore 117597 Singapore; ^9^ Theranostics Center of Excellence (TCE) Yong Loo Lin School of Medicine National University of Singapore 11 Biopolis Way, Helios Singapore 138667 Singapore; ^10^ Institute of Molecular and Cell Biology Agency for Science Technology, and Research (A*STAR) 61 Biopolis Drive, Proteos Singapore 138673 Singapore

**Keywords:** aortic aneurysm, extracellular matrix, fibroblast activation protein, positron emission tomography imaging

## Abstract

Traditional imaging modalities used to monitor the diameter of aortic aneurysms (AAs) often fail to follow pathological progression. Fibroblast activation protein (FAP), a key regulator of extracellular matrix (ECM) remodeling, plays a pivotal role in aortic disease. However, its expression in the aortic wall during aneurysm progression and its potential correlation with disease severity remains unexplored. Here, utilizing histology the levels of FAP are higher in the aortic wall of patients with AA compared to healthy controls. In three distinct animal models of AA, a progressive increase in FAP expression, coincides with the advancement of ECM remodeling. Notably, the levels of ^68^Ga‐FAPI‐04 uptake in a rabbit model of abdominal AA (AAA) is positively correlated with aortic dilation (*r* = 0.84, *p* < 0.01), and the histological examination further confirmed that regions of high ^68^Ga‐FAPI‐04 uptake exhibited both increased FAP expression and more severe pathological changes. The ^68^Ga‐FAPI‐04 imaging in AA patients showed that the radiotracer specifically accumulated in the aortic walls of persistently dilated AA. These findings suggest that ^68^Ga‐FAPI‐04 positron emission tomographic (PET) imaging, by visualizing FAP localization, allows for a non‐invasive approach to potentially monitor ECM remodeling during the AA progression.

## Graphical abstract

[^68^Ga]‐FAPI‐04 PET imaging technique represents a potential method to assess the extent of AA remodeling in vivo and may help to identify candidates at potential risk for rapid AA progression.

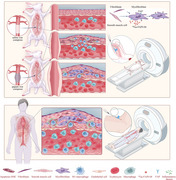

## 1 Introduction

Aortic aneurysms (AAs) are characterized by abnormal dilation of the aortic wall, which can cause fatal outcomes during the progression stage.^[^ ^1^ ^]^ Given the asymptomatic nature of AAs, computed tomography (CT) and magnetic resonance imaging (MRI) are the primary methods used to detect and monitor their progression.^[^ ^2^ ^]^ Surgical recommendations for patients with AAs are based on the aneurysm diameter and the rate of aortic growth.^[^ ^3^ ^]^ However, this approach may not be sufficient to differentiate the pathological variations within the wall of the aneurysm between patients at high risk of aortic rupture or dissection and those at lower risk during the dilation of AAs. Notably, the risk of aortic dissection or rupture within 5 years of ascending AA with diameters from 4.5 to 5.5 cm is 0.4% to 2.9%, respectively.^[^ ^4^ ^]^ The annual rupture rates of abdominal AA (AAA) with a diameter from 4.0 to 4.9 cm range from 0.5% to 5%, respectively, and 23% of ruptured AAA cases are less than 5 cm.^[^ ^5^ ^]^ Therefore, it is imperative to find new technologies that can identify pathological transformations to effectively monitor the pathological progression of AAs independently of changes in diameter.

In this context, positron emission tomography (PET) imaging has emerged as a potential method for more precise diagnosis and prognosis assessment of AAs. For example, fluoro‐2‐deoxy‐D‐glucose (FDG) can be taken up by activated inflammatory cells, which play an important role in the occurrence of AAs. However, the relationship between the uptake levels of ^18^F‐FDG in the AA wall and the progression of AAs during follow‐up remains controversial. A comparative study indicated that there was no significant difference in the number of patients with increased uptake of ^18^F‐FDG in an AAA group compared to a control group.^[^ ^6^ ^]^ Furthermore, a prospective study suggested that ^18^F‐FDG‐based PET is not an effective method for evaluating the dilation risk of small and asymptomatic AAA.^[^ ^7^ ^]^ In addition, studies in an animal model of AAA also found that the uptake level of ^18^F‐FDG was high in the early stage of AAA and gradually decreased during the aortic dilation.^[^ ^8^ ^]^ These findings indicate that the ^18^F‐FDG might not be a reliable biomarker for monitoring the progression of AAs.

AAs are generally divided into thoracic aortic aneurysms (TAA) and AAA. TAA and AAA share some common pathological characteristics, most notably reduced contractility, elastic capability, and ECM remodeling.^[^ ^9^ ^]^ ECM proteins play important roles in mediating the interaction between cells, as well as allowing cells to communicate with the extracellular microenvironment, and these proteins are responsible for the transmission of muscle fiber force and its maintenance and repair. However, under the influence of pathological mechanical forces, collagen degradation and abnormal synthesis, assembly, aggregation, and deposition of ECM proteins both contribute to the pathogenesis of TAA and AAA.^[^ ^10^ ^]^ During the ECM remodeling, the matrix metalloproteinases (MMPs), secreted by fibroblasts, vascular smooth muscle cells (VSMCs), and macrophages, e.g., play an important role in the collagen degradation both in TAA and AAA.^[^ ^11^ ^]^ Given the characteristics of MMPs in AA, many molecular imaging techniques have been developed to track the expression and activity of MMPs as indicators of disease progression.^[^ ^12^ ^]^ However, there are few studies on the use of these tracers in AAA. Additionally, the MMP family consists of 28 members, at least 23 of which are expressed in human tissues, of which MMP −1, −2, −3, −9, −12, and −13 are associated with AAs.^[^ ^13^ ^]^ This complexity may limit their application in the monitoring of AA progression. Therefore, it is crucial to identify new molecular imaging targets that can specifically reflect the extent of ECM remodeling during the progression of AAs.

**Figure 1:**
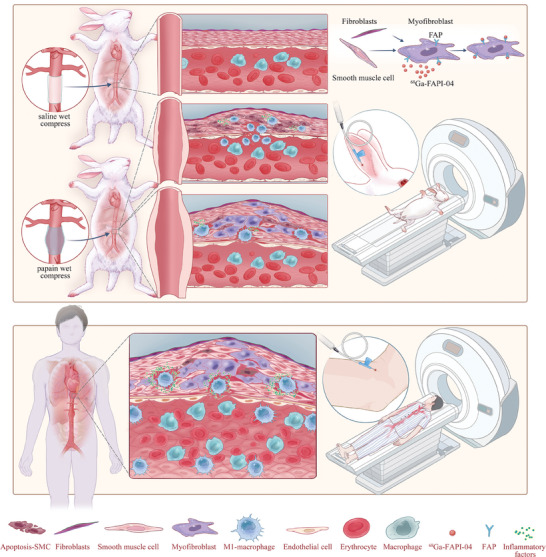
The mechanism by which ^68^Ga‐FAPI‐04 PET/MR imaging can evaluate AA progression. A diagram illustrating that in the New Zealand rabbit model of AA, the FAP‐expressing activated myofibroblasts continually increase during AA expansion. ^68^Ga‐FAPI‐04‐based imaging in New Zealand rabbits AA can reflect the FAP levels and indirectly reflect the expression of FAP^+^ myofibroblasts by targeting FAP, showing a positive correlation between the uptake levels of ^68^Ga‐FAPI‐04 and ECM remodeling (upper part of the picture). ^68^Ga‐FAPI‐04 exhibits a higher uptake in AA patients with a history of persistent aortic dilation (lower part of the picture). ECM, extracellular matrix.

Fibroblast activation protein (FAP) is a prolyl‐specific serine protease and is involved in the ordered proteolysis of collagen and matrix remodeling. It has been utilized to evaluate the ECM degradation in cancer to reflect the occurrence of tumor metastasis.^[^ ^14^ ^]^ Under normal physiological conditions, FAP levels remain low but are upregulated in cells involved in ECM remodeling, demonstrating strong specificity.^[^ ^15^ ^]^ Additionally, FAP inhibitor (FAPI) has been designed to specifically target FAP and can be radiolabeled to determine FAP expression in vitro,^[^ ^16^ ^]^ and FAPI‐directed PET has been explored as a method to assess FAP levels in vivo.^[^ ^17^ ^]^ Therefore, we hypothesized that FAP could also serve as a biomarker to monitor ECM remodeling during the progression of AAs. Here, we aimed to utilize ^68^Ga‐FAPI‐04‐based PET imaging to visualize FAP levels in both animal models and human cases of AA and to ascertain the correlation between FAP expression and ECM remodeling in AAs (**Figure** **1**).

## 2 Results and Discussion

### 2.1 Upregulated FAP Expression in Aortic Aneurysms Is Associated with Myofibroblasts and Aortic Diameter

**Figure 2:**
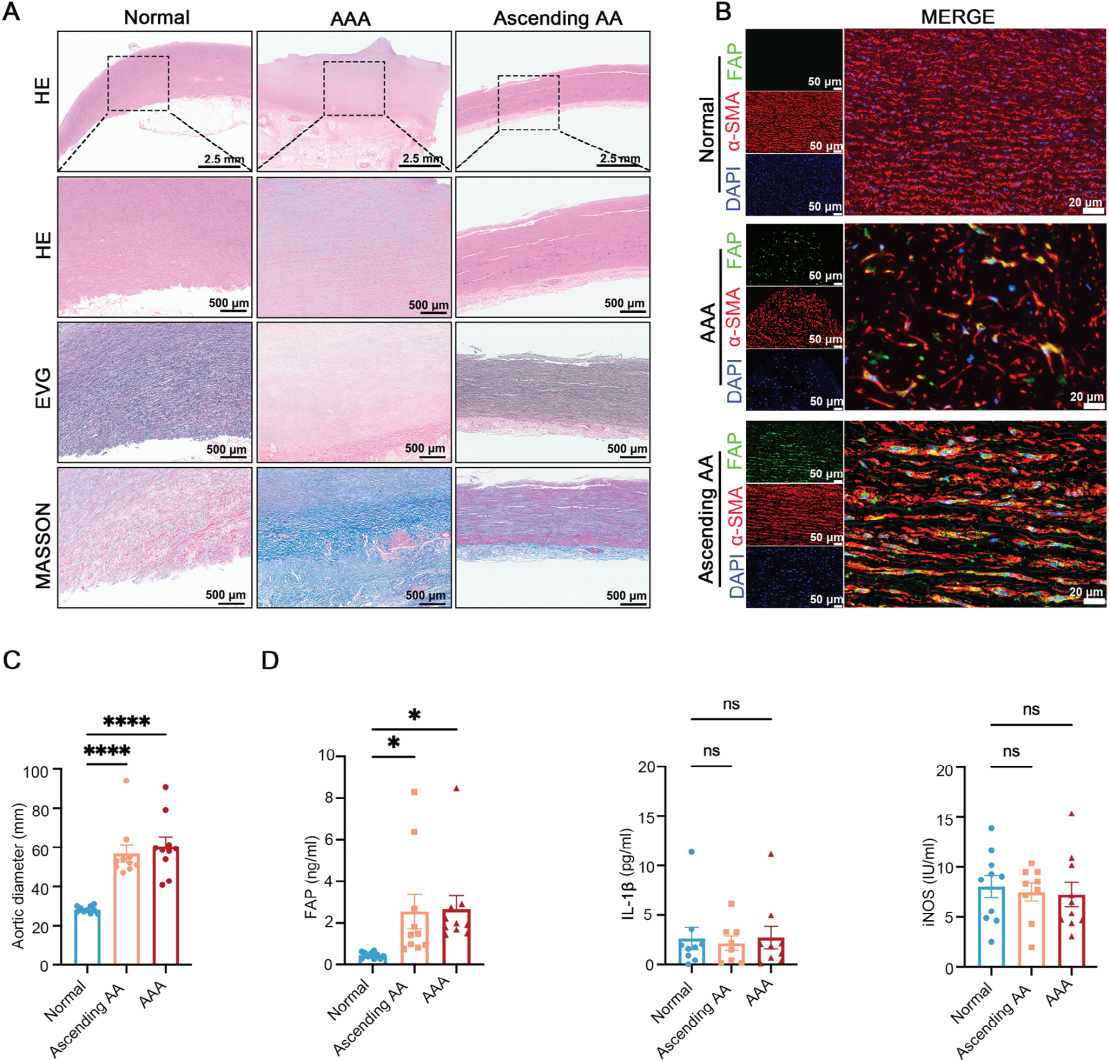
The histological examination and ELISA indicate a correlation between FAP and AA. A) Histology of normal ascending aorta, AAA, and ascending AA samples. Scale bar = 500 µm. B) Immunofluorescence staining of normal ascending aorta, AAA, and ascending AA samples. The co‐staining of FAP and α‐SMA was used to reflect FAP^+^ myofibroblasts. Scale bar = 500 µm. C,D) The aortic diameter and ELISA results of FAP, IL‐1b, and iNOS in AA patients and healthy volunteers. AAA, abdominal aortic aneurysm; FAP, fibroblast activation protein.

Histological analysis of human samples revealed that healthy controls exhibited an intact structure of the aortic walls, while individuals with AAA or ascending AA showed clear evidence of elastic destruction and collagen deposition (**Figure** **2**A). Moreover, the immunofluorescence staining exhibited a higher co‐localization of FAP and α‐smooth muscle actin (α‐SMA) expression in aortic samples from individuals with AAA and ascending AA compared to normal controls (Figure 2B), suggesting that FAP may be correlated with myofibroblasts activation. Myofibroblasts are a specialized cell population that originates under pathophysiological conditions, primarily involved in ECM production and remodeling, secretion of angiogenic and pro‐inflammatory factors, and generation of contractile forces.^[^ ^18^ ^]^ The high expression of FAP in the wall of AA indicates that the FAP may play an important role in the progression of AA, but the specific mechanism still needs further study.

Given that FAP is a secreted protein, we subsequently collected plasma samples from individuals with ascending AA (*n* = 10) or AAA (*n* = 10) as well as from normal volunteers (*n* = 10) (Figure 2C). The baseline characteristics are shown in Table S1 (Supporting Information). The ELISA results showed that plasma FAP levels were higher in patients with ascending AA and AAA compared to normal controls (Figure 2D). However, there were no significant differences in iNOS and IL‐1β levels between the patients with the two types of AA and the normal volunteers (Figure 2D). A 5‐year follow‐up study of 206 patients with AAA also revealed that inflammatory markers, such as tumor necrosis factor (TNF)‐α and interleukin (IL)‐6, did not significantly change during aortic diameter growth.^[^ ^19^ ^]^ These findings suggest that inflammatory markers may have poor sensitivity in the monitoring of AAA progression during follow‐up.^[^ ^20^ ^]^

### 2.2 Temporal Dynamics of FAP Expression and ECM Remodeling in the BAPN‐Induced TAA Model

**Figure 3:**
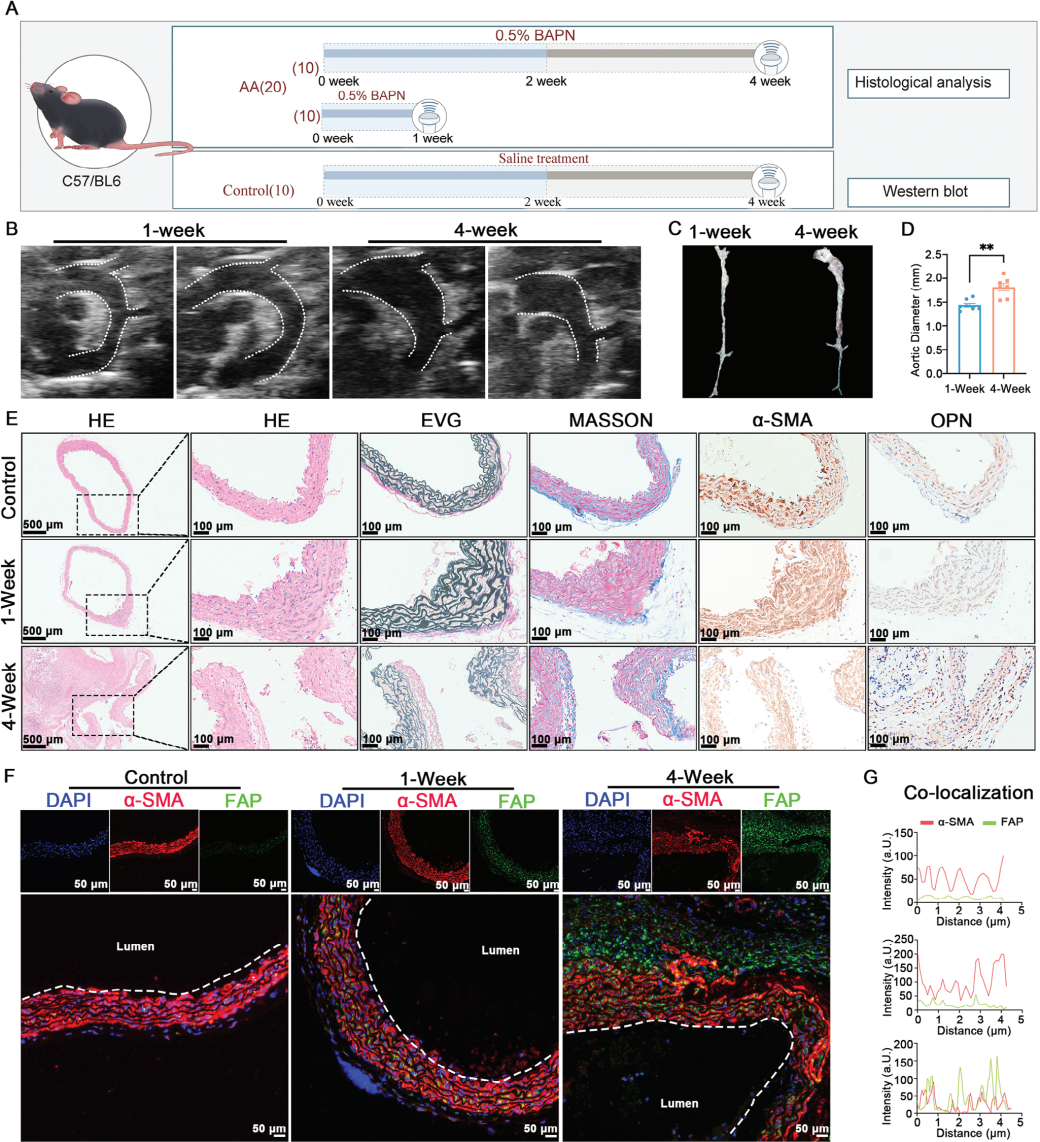
The BAPN‐induced TAA model exhibits a positive correlation between the levels of FAP and aortic remodeling. A) A schematic diagram illustrating the experimental design and analyses of the BAPN‐induced TAA model in mice. B,C) ultrasonic examination (B) and gross morphology (C) of aortas in the indicated groups. D) The aortic diameter of the indicated groups (1‐week, *n* = 7; 4‐week, *n* = 7). E) H&E, EVG, and Masson staining and immunohistochemical expression of α‐SMA and OPN in specimens from the indicated groups of the BAPN‐induced TAA model (control, *n* = 4; 1‐week, *n* = 4; 4‐week, *n* = 4). Scale bars = 100 µm. F) Immunofluorescence staining, investigating the co‐expression of FAP and α‐SMA, of the indicated groups of BAPN‐induced TAA mice. Dashed lines mean the inner wall of the artery. Scale bar = 50 µm. G) Co‐localization analysis of FAP and α‐SMA in the medial layer of the aorta among the indicated groups of the BAPN TAA model (from upper to lower indicated the control, 1‐week, and 4‐week). The red line indicates α‐SMA expression, while the green line represents FAP expression. ^*^ *p* < 0.05, ^**^ *p* < 0.01, and ^***^ *p* < 0.001, ns, not statistically significant.

To investigate the trend of changes in FAP expression during the TAA development, we induced TAA in 3‐week‐old wild‐type C57/BL6 male mice by treating them with 0.5% β‐aminopropionitrile (BAPN) (wt/vol) in the drinking water for 1 or 4 weeks. The administration of BAPN, a commonly used inducer of TAA and thoracic aortic dissection, leads to primary histological changes consistent with AA after 7 days of intervention.^[^ ^21^ ^]^ By ultrasonic examination, we found a significantly greater mean aortic diameter in the 4‐week‐treated group compared to the 1‐week‐treated group (1.8 mm versus 1.4 mm, *p* < 0.001) (**Figure** **3**A‐D). Histological analyses of aortic tissue samples revealed notable progressive pathological changes in the 4‐week‐treated group (Figure 3E). Immunofluorescence staining of FAP and α‐SMA showed that during the AA induction process, the expression of FAP gradually increased (Figure 3F). Additionally, the co‐localization of these two markers showed a greater degree of overlap in the 4‐week‐treated group compared to the 1‐week‐treated group, indicating an increase in FAP^+^ myofibroblasts over time, which is associated with TAA progression (Figure 3G). Furthermore, we observed that some FAP did not colocalize with α‐SMA, suggesting that FAP may originate from a variety of cells, such as myofibroblasts and activated fibroblasts.^[^ ^15^, ^22^ ^]^ Therefore, exploring the functions of FAP in different cell types in future studies may help to further elucidate its potential mechanism in the ECM remodeling during the progression of AA.

### 2.3 Similar Trends of FAP Expression during ECM Remodeling Are Observed in the Ang II‐Induced AAA in ApoE^−/−^ Mice

**Figure 4:**
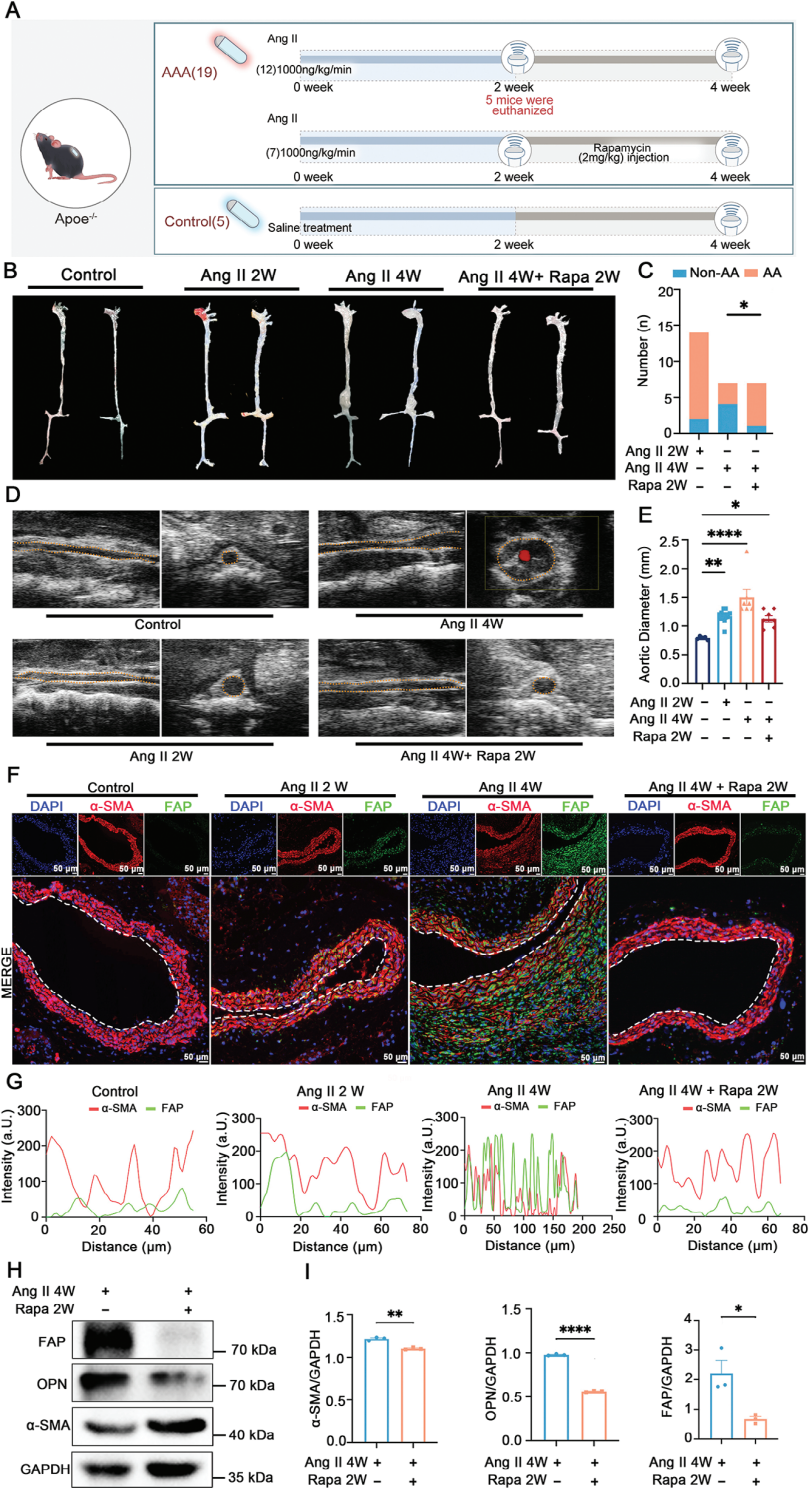
The Ang II‐induced AAA model in *ApoE* ^−/−^ mice demonstrates the potential of FAP as a prognostic marker for AAA. A) A schematic diagram illustrating the experimental design and analysis of the Ang II AAA model in *ApoE^−/−^* mice. B,C) Gross anatomy (B) and the quantification of the aortic aneurysm (AA) regions versus non‐AA regions (C) from the indicated groups. D,E) The ultrasonic examination of aortas (D) and the quantification of aortic diameters (E) from the indicated groups (control, *n* = 5; Ang II 2w, *n* = 14; Ang II 4w, *n* = 7; Ang II 4w + Rapa 2w, *n* = 7). F) Immunofluorescence examinations of α‐SMA (red) and FAP (green) in specimens from the indicated groups of *ApoE* ^−/−^ mice (control, *n* = 3; Ang II 2w, *n* = 4; Ang II 4w, *n* = 4; Ang II 4w + Rapa 2w, *n* = 4). Dashed lines mean the inner wall of the artery. Scale bar = 50 µm. G) Co‐localization analysis of FAP and α‐SMA in the medial layer of aorta among the indicated groups of *ApoE^−/−^* mice. The red line indicates α‐SMA expression, while the green line represents FAP expression. H,I) Western blot analysis (H) and its quantification (I) of the aorta tissues from the indicated groups of *ApoE* ^−/−^ mice (Ang II 4w, *n* = 3; Ang II 4w + Rapa 2w, *n* = 3). The data are presented as the mean ± SD. ^*^ *p* < 0.05, ^**^ *p* < 0.01, and ^***^ *p* < 0.001, ns, not statistically significant. Rapa, Rapamycin.

The Angiotensin II (Ang II)‐induced AAA model in *ApoE^−/−^* mice is another widely used animal model of AA. Rapamycin, a classical mTOR inhibitor, has been shown to attenuate AA progression in vitro and in vivo.^[^ ^23^ ^]^ In this study, we induced AAA by administering 1000 ng kg^−1^ min^−1^ of Ang II via an osmotic pump in 8‐week‐old *ApoE* ^−/−^ male mice to explore the temporal change levels of FAP during the AAA progression and determined whether rapamycin (2.0 mg kg^−1^) could mitigate FAP levels (**Figure** **4**A).

Our results showed that co‐treatment of rapamycin inhibited AA progression compared with *ApoE* ^−/−^ mice treated with Ang II alone for 4 weeks (Figure 4B,C). The echocardiography analyses exhibited that the mean diameter of the abdominal aorta in the Ang II‐treated group continued to increase from 2 weeks to 4 weeks, while it was narrower in the group of mice treated with Ang II for 4 weeks combined with rapamycin treatment for 2 weeks [1.13 mm (Ang II + Rapamycin treated) versus 1.5 mm (Ang II treated), *P* = 0.005] (Figure 4D,E).

Histological examinations of aortic samples revealed ongoing degradation of elastic fibers, collagen deposition, and elevated OPN (a marker of synthetic VSMCs) expression during the Ang II intervention, which was alleviated in the rapamycin‐treated group (Figure S1, Supporting Information, we observed). These findings are consistent with a previous study.^[^ ^24^ ^]^ Immunofluorescence staining of translocator protein (TSPO), a marker of macrophage activity, indicated more inflammatory cells in the aortic wall at 2 weeks compared to 4 weeks of Ang II treatment (Figure S2, Supporting Information), suggesting that there was no further increase in inflammatory cell infiltration during AAA progression. Immunofluorescence staining of FAP and α‐SMA also showed that during Ang II induction, the expression of FAP gradually increased, and this trend was reversed in mice treated with Ang II for 4 weeks combined with rapamycin for 2 weeks (Figure 4F). The co‐localization of the FAP and α‐SMA exhibited a gradual elevation in the number of FAP^+^ myofibroblasts within the aortic wall from 2 weeks to 4 weeks of Ang II treatment, which was reduced by the 2‐week rapamycin treatment (Figure 4G). The co‐localization of FAP and α‐SMA was observed not only in the medial wall but also in the adventitial wall, indicating that myofibroblasts may be derived from both VSMCs and fibroblasts, consistent with related studies.^[^ ^18^ ^]^ However, further experiments are required to verify these findings.

Western blot analyses of aortic samples from *ApoE^−/−^* mice treated with Ang II for 4 weeks and those treated with Ang II for 4 weeks combined with 2 weeks of rapamycin corroborated the histological observations, showing lower FAP and OPN expression and higher α‐SMA expression following rapamycin treatment (Figure 4H,I). These results suggest that FAP levels in the aortic wall could be a potential biomarker of pathological changes during the development of AA.

To further explore the changes of differentially expressed genes (DEGs) in *ApoE^−/−^* AAA model, we selected microarray dataset GSE17901 of the suprarenal aorta of *ApoE^–/–^* mice after Ang II infusion for 7 days, 14 days, and 28 days from the GEO database. Upon intersecting the differentially expressed genes (DEGs) from the three groups, we identified a total of 101 common DEGs (Figure S3A, Supporting Information). Among the 101 common DEGs, we found that Fap and related collagen genes such as *Col5a1*, *Col8a1*, *Col8a2*, and *Col11a1*, were consistently higher in expression within the aorta from 7 days to 14 days and 28 days (Figure S3B, Supporting Information). Gene ontology (GO) analysis of the common DEGs revealed significant terms related to the collagen‐containing extracellular matrix (Figure S3C, Supporting Information). Kyoto Encyclopedia of Genes and Genomes (KEGG) analysis indicated that the common DEGs were mainly enriched in the protein digestion and absorption signaling pathway (Figure S3D, Supporting Information). These findings indicate that the presence of FAP could serve as a potential indicator for monitoring the temporal trends of ECM remodeling during AAA development. However, more studies are still needed to explore the role of FAP expression in different cell types.

### 2.4 PET/CT Imaging Facilitates Temporal Analysis of Tissue FAP Levels in a Rabbit Model of AAA

**Figure 5:**
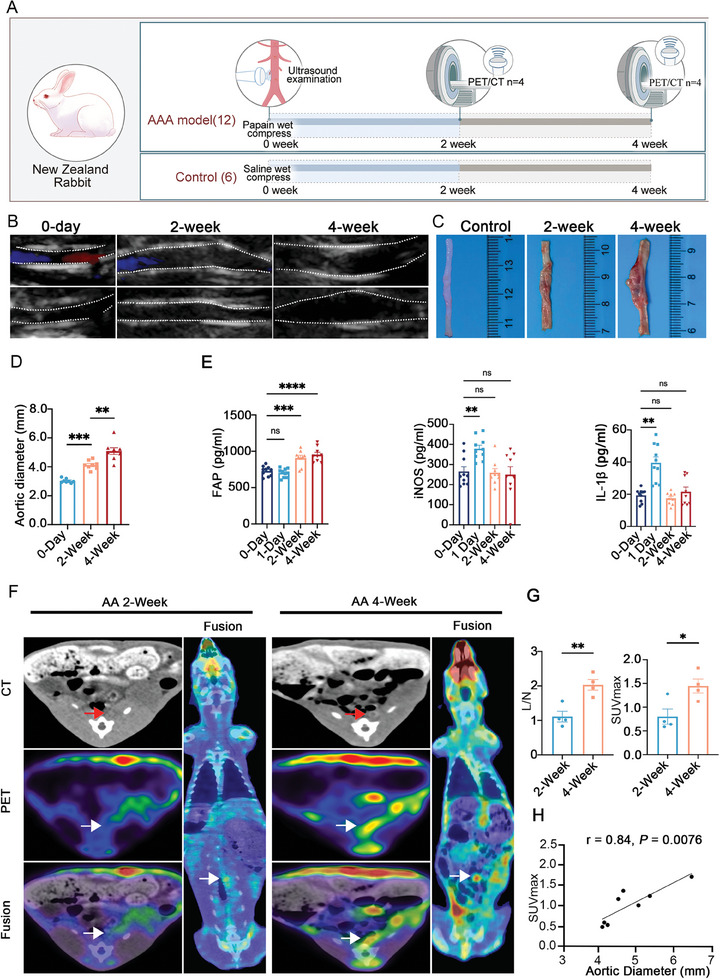
^68^Ga‐FAPI‐04 PET/CT imaging and plasma biomarkers testing in rabbits AAA. A) A schematic diagram illustrating the experimental design of the rabbit AAA model and its analysis. B,C) ultrasonic (B) and the gross anatomy (C) of the aorta in the indicated groups. D) The diameter of the abdominal aorta in the indicated groups of New Zealand rabbits. E) Plasma biomarkers levels, including FAP, iNOS, and IL‐1β, in the indicated groups of New Zealand rabbits. F,G) Imaging by ^68^Ga‐FAPI‐04‐based PET/CT scanning (F) and its quantitation (G) of New Zealand rabbits at 2‐ and 4‐week points of post‐operation (Red arrows indicate the aorta; white arrows indicate the high uptake of ^68^Ga‐FAPI‐04 sites). H) A correlation analysis between SUVmax levels and AAA diameter. The data are presented as the mean ± SD. *Ρ < 0.05, **Ρ < 0.01, and ***Ρ < 0.001, ns, not statistically significant.

The *ApoE^−/−^* AA mice model predominantly induces AAA in the suprarenal aorta, which differs from human AAA, which mainly occurs in the infrarenal abdominal aorta.^[^ ^25^ ^]^ To observe the temporal changes in the levels of FAP during AA development in *vivo*, we established an infrarenal AAA model in rabbits to better mimic human AAA progression by performing partial ligation of the left renal artery combined with local application of papain (**Figure** **5**A). The results of ultrasonic and anatomy revealed consistent dilation of the infrarenal aortic wall from post‐operation to 4 weeks (Figure 5B,C). The mean aortic diameter in the papain‐treated area gradually increased over time: [0.299 cm (0‐day) versus 0.411 cm (2‐week), *p* = 0.0002; 0.411 cm (2‐week) versus 0.510 cm (4‐week), *p* = 0.003] (Figure 5D). Interestingly, the ELISA analysis of plasma FAP levels showed a significant increase from preoperative levels to those at the 4‐week post‐operation point (728.8 pg mL^−1^ versus 953.1 pg mL^−1^, P < 0.001) (Figure 5E), with a correlation between plasma FAP levels and aortic diameter in the rabbit AAA model (*r* = 0.66, *p* < 0.01) (Figure S4, Supporting Information). Furthermore, four New Zealand rabbits were randomly selected for ^68^Ga‐FAPI‐04 PET/CT imaging at the 2‐week and the 4‐week post‐operation time points. Comparing the outcomes between those two‐time points revealed a higher uptake of ^68^Ga‐FAPI‐04 in the infrarenal abdominal aorta at the 4‐week time point, displaying high specificity and consistency with the papain‐treated site (Figure 5F). The lesion/normal pulmonary artery site (L/N) ratio was also higher in the 4‐week point imaging compared to the 2‐week point imaging (2.032 versus 1.113, *p* = 0.006) (Figure 5G). Additionally, there was a positive correlation between the uptake of ^68^Ga‐FAPI‐04 levels and aortic diameter in the New Zealand rabbit AAA model (*r* = 0.84, *p* = 0.0076) (Figure 5H).

**Figure 6:**
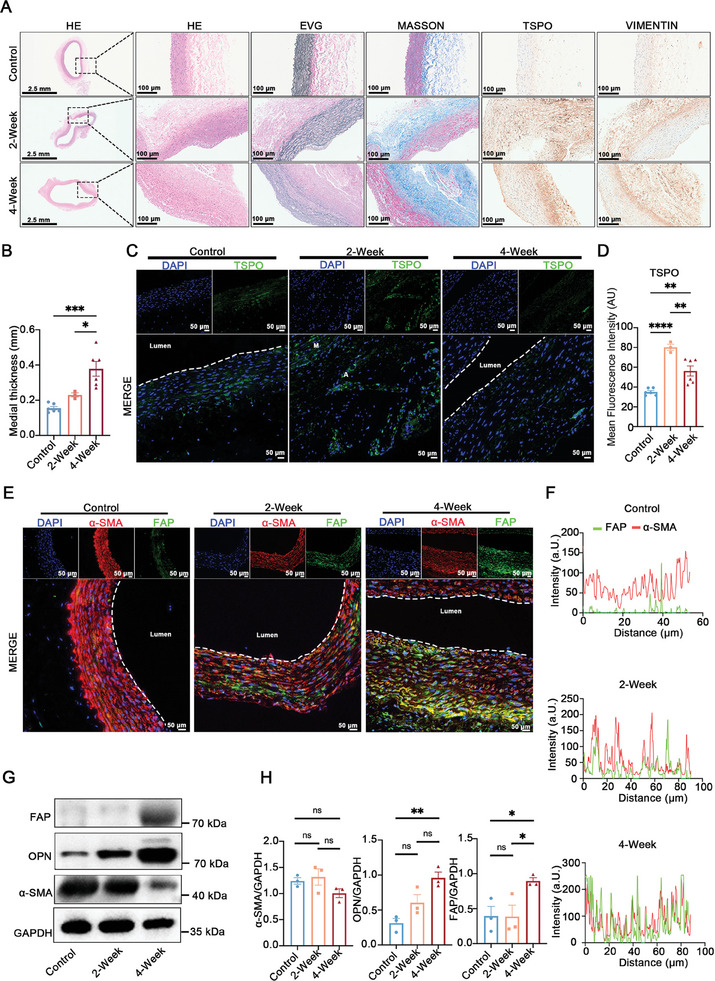
Histological and western blot analyses confirm the temporal changes of tissue FAP levels during AAA progression. A) The outcomes of H&E, EVG, Masson blue staining, and immunohistochemical examinations of TSPO and vimentin of specimens from the indicated groups of New Zealand rabbits. B) The medial thickness of aortic specimens from the indicated groups of New Zealand rabbits. C,D) Immunofluorescence analysis (C) and its quantification (D) of TSPO (green) from the indicated groups of New Zealand rabbits. E) Immunofluorescence analysis, investigating the co‐expression of FAP and α‐SMA, among the indicated groups of New Zealand rabbits. F) Co‐localization analysis of FAP and α‐SMA in the medial layer of the aorta among the indicated groups of New Zealand rabbits. The red line indicated α‐SMA expression, while the green line represented FAP expression. G,H) The western blot of FAP, OPN, and α‐SMA (G) and its quantification (H) in the indicated groups of New Zealand rabbits. ^*^ *p* < 0.05, ^**^ *p* < 0.01, and ^***^ *p* < 0.001, ns, not statistically significant. TSPO, Translocator protein.

The histological examination of rabbit aortic tissue samples from control, 2‐week, and 4‐week time points revealed a progressive pattern of AAA development, characterized by continuous disruption of elastic fibers and collagen deposition (**Figure** **6**A). Compared to the 2‐week AAA, the 4‐week AAA had greater mean medial thickness (0.22 mm versus 0.33 mm, *p* = 0.007, Figure 6B). Immunofluorescence analysis of TSPO showed that inflammatory cell infiltration was more pronounced at the 2‐week stage of AAA compared to the 4‐week stage (mean fluorescence intensity 80.1 versus 56.2, *p* = 0.005) (Figure 6C,D). Furthermore, immunofluorescence staining for FAP and α‐SMA demonstrated a gradual increase in FAP expression from normal rabbits to the 2‐week and then to the 4‐week time points (Figure 6E). Moreover, the co‐localization of the two markers revealed a greater degree of overlap in the 4‐week time point compared to the 2‐week time point (Figure 6F). Additionally, the distribution of FAP expression in the aortic wall was similar to that observed in the *ApoE^−/−^* AAA mouse model. Finally, western blot analysis of aortic samples showed a continuous increase in FAP expression over time, corroborating the immunofluorescence staining results (Figure 6G,H).

These findings demonstrate that the ^68^Ga‐FAPI‐04 PET/CT imaging can effectively reflect the level of FAP in the AAA wall, and the aortic wall with higher uptake levels of FAP showed more elastic fibers degradation, collagen deposition, and larger aortic diameter. However, more studies are necessary to validate the correlation between FAP levels and AA dilation.

### 2.5 Validation of FAP Levels in in patients with AA Through ^68^Ga‐FAPI‐04 PET/MR Imaging

After obtaining informed consent, six patients diagnosed with AA underwent ^68^Ga‐FAPI‐04‐based PET/MR imaging to assess FAP levels in the aortic wall. The characteristics and details of patients are shown in Table S2 (Supporting Information).

**Figure 7:**
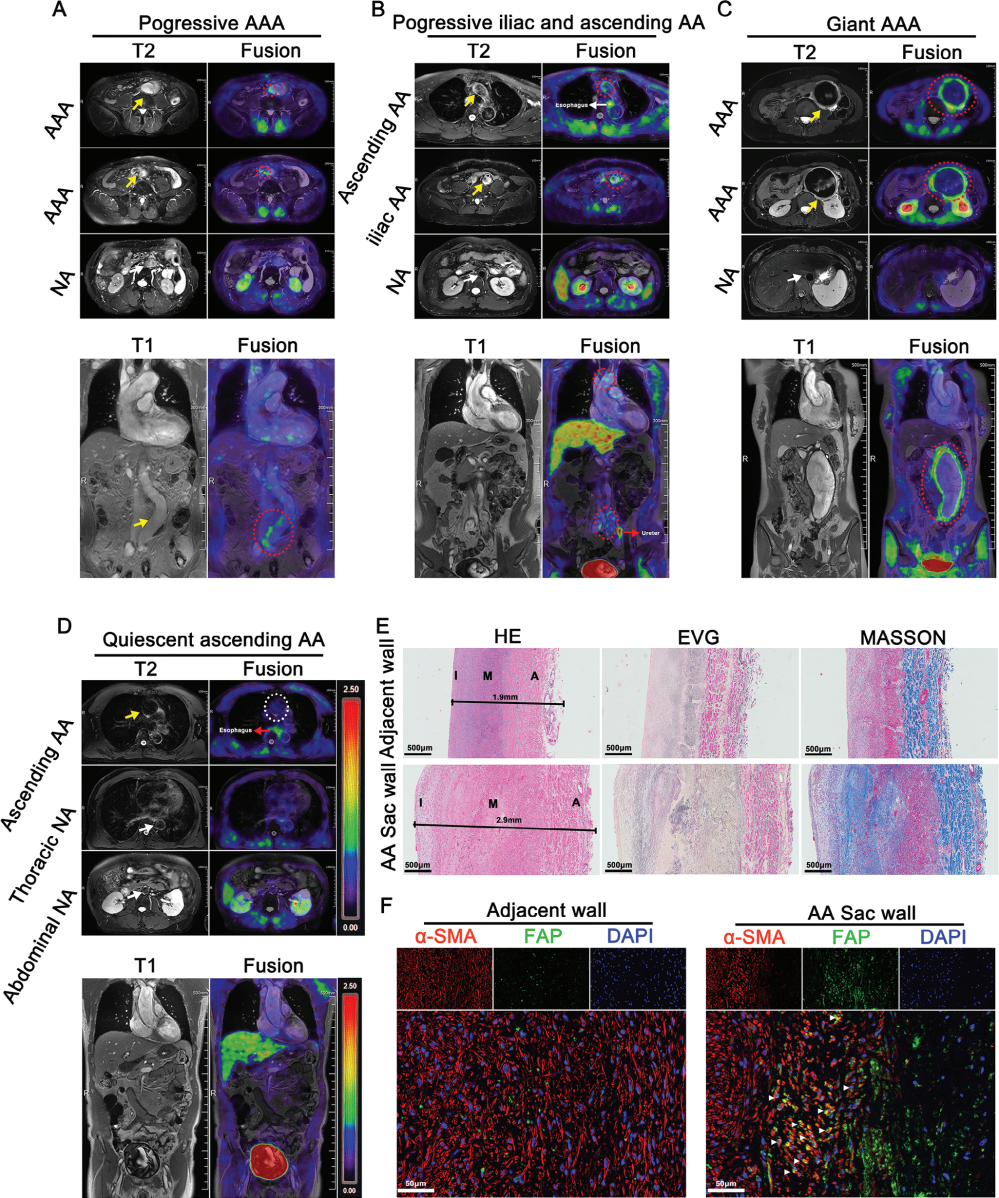
^68^Ga‐FAPI‐04 PET/MR imaging in AA patients showed that the aortic wall with a tendency to dilation had higher FAP uptake. A) ^68^Ga‐FAPI‐04 PET/MR imaging of a patient with progressive abdominal aortic dissecting aneurysm (red circles indicate the ^68^Ga‐FAPI‐04 uptake in the AA wall, yellow arrow indicates the AA and white arrow indicates the normal aorta). B) ^68^Ga‐FAPI‐04 PET/MR imaging of a patient with progressive iliac aortic dissecting aneurysm and ascending AA (red circles indicate the ^68^Ga‐FAPI‐04 uptake in the AA wall, yellow arrow indicates the AA and white arrow indicates the normal aorta). C) ^68^Ga‐FAPI‐04 PET/MR imaging of a patient with giant AAA (red circles indicate the ^68^Ga‐FAPI‐04 uptake in the AA wall, yellow arrow indicates the AA and white arrow indicates the normal aorta). D) ^68^Ga‐FAPI‐04 PET/MR imaging of a patient with a quiescent period of ascending AA (white circle indicates no ^68^Ga‐FAPI‐04 uptake in the AA wall, yellow arrow indicates the AA and white arrow indicates the normal aorta). E) H&E, EVG, and Masson blue staining of specimens from the third patient's AA sac wall and adjacent wall. F) Immunofluorescence of α‐SMA (red) and FAP (green) in specimens from the AA sac wall and adjacent wall (white triangles indicate positive cells). Scale bar = 50 µm. NA, normal aorta.

In the first case, a patient with an aortic dissecting aneurysm in the abdominal aorta exhibited a consistent increase in aortic diameter by 0.1 cm per year over the past five years. In this patient, anomalous uptake of ^68^Ga‐FAPI‐04 was observed exclusively in the wall of AA, with a target‐to‐background ratio (TBR) of 2.27, expressed by the following equation [TBR = Max(VOI_lesion_)/ Mean(VOI_Blood_)] (**Figure** **7**A). Similarly, in the second case, outpatient CT angiography (CTA) performed prior to ^68^Ga‐FAPI‐04 PET/MR imaging showed that the diameters of 48.5 mm for the ascending AA and 35 mm for the iliac AA. During a 1‐year follow‐up, the diameters of the ascending AA and iliac AA increased to 49.6 and 37.2 mm, respectively (Figure S5, Supporting Information). ^68^Ga‐FAPI‐04‐based imaging detected abnormal uptake of ^68^Ga‐FAPI‐04 in the ascending AA with a TBR of 1.94 and in the iliac AA with a TBR of 2.6 (Figure 7B). The third case had a giant AAA with a diameter of 81 mm, accompanied by abdominal pain. ^68^Ga‐FAPI‐04‐based imaging showed obvious abnormal uptake of the ^68^Ga‐FAPI‐04 in the AAA with a TBR of 4.21 (Figure 7C). The fourth case had an AAA with an aortic diameter increase of 0.2 cm per year over the previous year, and the ^68^Ga‐FAPI‐04‐based imaging revealed an abnormal uptake of ^68^Ga‐FAPI‐04 in the AAA with a TBR of 2.57. The fifth case involved an ascending AA with no significant dilation over the previous three years. ^68^Ga‐FAPI‐04‐based imaging revealed no abnormal uptake in the ascending AA with a TBR of 1.22 (Figure 7D). The sixth case also had a quiescent ascending AA, and ^68^Ga‐FAPI‐04‐based imaging revealed no abnormal uptake in the ascending AA with a TBR of 1.06.

As the maximum diameter of the AAA was 81 mm, the third patient underwent aortic replacement. Histological examination of the AA sac wall with high ^68^Ga‐FAPI‐04 uptake and the adjacent wall with low uptake showed more elastic degradation and collagen deposition in the AA sac wall (Figure 7E). Immunofluorescence staining of FAP and α‐SMA also demonstrated that the AA sac wall with high ^68^Ga‐FAPI‐04 uptake had greater expression of FAP and FAP^+^ myofibroblasts than the adjacent wall (Figure 7F).

These findings suggest that the ^68^Ga‐FAPI‐04 PET imaging can effectively reflect the levels of FAP in human AA. Additionally, the aortic walls with higher uptake levels of ^68^Ga‐FAPI‐04 exhibited more disruption of elastic fibers and collagen deposition, suggesting that ^68^Ga‐FAPI‐04 imaging has the potential advantage of reflecting the ECM remodeling during AA progression. However, more clinical studies are necessary to verify this hypothesis.

## 3 Conclusion

FAP expression continues to increase with the AA development suggesting it may be a critical role during the development of AA. ^68^Ga‐FAPI‐04‐based imaging demonstrates high specificity in detecting FAP expression in AA, with higher uptake levels of ^68^Ga‐FAPI‐04 corresponding to greater disruption of elastic fibers and collagen deposition in the aortic wall. Thus, this imaging technique represents a potential means to assess the ECM remodeling during the progression of AA in vivo.

## 4 Experimental Section

### Human Samples

All human research was conducted in accordance with official ethical guidelines and approved by the Ethics Committee of the Zhong Shan Hospital of Fudan University (Approval NO: B2022‐098R2). Written informed consent was obtained from all participants prior to sample collection. AA samples were collected from patients undergoing aortic replacement procedures, while control aortic samples were sourced from individuals who underwent a heart transplant. For the plasma FAP testing, patients diagnosed with ascending AA and AAA were recruited from the cardiac vascular surgery department. Healthy controls were recruited from the physical examination center. Echocardiograms and blood samples were obtained from all participants to assess the correlation between plasma FAP levels and aortic diameter.

### AA Model Experiments

In this study, three AA animal models were established to explore the correlation between FAP and AA progression. All animals feed in a normal environment and have free access to water and food. The relevant experiments were approved by the institutional committee of Zhongshan Hospital of Fudan University and Fudan University Affiliated Xuhui Hospital (Number: SOP‐IEC‐003‐02.0‐AF05).

### Mice—*Wild‐Type Mouse AA Model*

Three‐week‐old C57BL/6J male mice were purchased from Cyanogen company and randomly divided into three groups: a control group (*n* = 10), a 1‐week treatment group (*n* = 10), and a 4‐week treatment group (*n* = 10). The control group was fed a normal chow diet and regular drinking water, while the 1 and 4‐week groups were fed a normal chow diet and regular drinking water supplemented with 0.5% β‐Aminopropionitrile (BAPN, purchased from Sigma) wt/vol. After 1 week of BAPN treatment, mice in the 1‐week group underwent echocardiography examination under gas‐induced anesthesia. Following the recording of the diameter of the aorta, mice were euthanized, and aortic specimens were collected for histological testing. Similarly, after 4 weeks of feeding with BAPN, mice in the 4‐week group underwent echocardiography examination under gas‐induced anesthesia. Following the recording of the diameter of the aorta, mice in the control and 4‐week groups were euthanized, and aortic specimens were obtained for immunohistochemical testing. The relevant processes are illustrated in Figure 3A.

### Mice—*ApoE^−/−^ Mouse AAA Model*

Eight‐week‐old *ApoE* ^−/−^ male mice, purchased from Cyagen company, were randomly assigned to two groups: a control group (*n* = 5) and an Angiotensin II (Ang II, purchased from MedChenExpress corporate) group (*n* = 19). The control group was fed a regular chow diet and drinking water, while the Ang II group was treated with Ang II (purchased from MedChenExpress) at 1000 ng kg^−1^ min^−1^ by a subcutaneous injection through an ALZET osmotic pump to induce AAA. Two weeks following the Ang II administration, mice in the Ang II group underwent an echocardiography examination under gas‐induced anesthesia. After measuring the aortic diameter, 5 mice in the Ang II group were euthanized and the other 7 mice in the Ang II group were intraperitoneally injected with 2.0 mg kg^−1^ rapamycin (purchased from MedChenExpress) every other day for two weeks. Two weeks after rapamycin treatment, mice in both the control and Ang II groups underwent an echocardiography examination. Following recording the diameter of the aorta, all mice were euthanized, and aortic specimens were taken for histological and Western blot testing. The relevant processes are illustrated in Figure 4A.

### Mice—*Transcriptional Profiling Analysis of the Ang II‐Induced ApoE^−/−^ AAA*

The expression profile from the microarray dataset GSE17901 was retrieved from the GEO database,^[^ ^26^ ^]^ and batch effects were mitigated using the R package “limm”. Subsequently, the “limm” package was employed to identify differentially expressed genes (DEGs) in the aorta from samples treated with Ang II compared to saline‐treated controls. Genes were classified as DEGs if they met the criteria of an unadjusted *p*‐value < 0.05 and a |logFC| > 1. DEGs were processed separately for the 7‐, 14‐, and 28‐day time points and utilized the R package “ggplot” to generate Venn diagrams and volcano plots. To elucidate the role of these DEGs in AAA pathogenesis, Gene Ontology (GO) and Kyoto Encyclopedia of Genes and Genomes (KEGG) pathway analyses were conducted using the “cluster profile” R package, with a threshold of *p*‐value < 0.05 for statistical significance.

### Rabbits—*New Zealand Rabbit AAA Model*

Eighteen New Zealand male rabbits, averaging 2.0 kg in weight, were purchased from the HANGZHOU QIZHEN laboratory. The rabbits were randomly assigned to a control group (*n* = 6) and an AAA (*n* = 12) group. AAA was induced by partial ligation of the left renal artery combined with local application of papain, a powerful proteolytic enzyme that can degrade elastic fibers, in the distal section of the renal artery for 20 min. Prior to the operation, rabbits in the AAA group were subjected to intraperitoneal anesthesia with a hydrated chlorine chamber (4‐5 mL kg^−1^) for echocardiography examination and venous blood collection for ELISA. On day 1 after the operation, venous blood of the AAA group was collected for ELISA. Two weeks after the operation, rabbits in the AAA group underwent an echocardiography examination and venous blood collection for ELISA. After echocardiography examination, 4 rabbits in the AAA group were randomly selected for ^68^Ga‐FAPI‐04 PET/CT, and another 3 rabbits in the AAA group were randomly selected for sacrifice. At the 4‐week point of post‐operation, rabbits in the AAA group underwent echocardiography examination and venous blood collection for ELISA. The previously selected 4 rabbits underwent ^68^Ga‐FAPI‐04 PET/CT again. Finally, rabbits in the control and AAA groups were euthanized, and aortic specimens were obtained for histological and Western blot examination.

### Rabbits—*^68^Ga‐FAPI‐04 PET/CT Imaging in the Rabbits*

The above four rabbits were selected for PET/CT imaging at a 2‐week time point post‐operation and received an injection of ^68^Ga‐FAPI‐04 at a dose of 27.75 Mbq kg^−1^. All imaging was performed using a clinical PET/CT scanner (Biograph64, Siemens Healthcare, Erlangen, Germany). 1‐h post‐injection, a PET scan was conducted using a 3D mode, and CT images were acquired under intraperitoneal anesthesia. At a 4‐week time point post‐operation, these four rabbits received ^68^Ga‐FAPI‐04 PET/CT imaging again following the above process. To quantify tracer uptake, circular regions of interest (ROIs) were positioned around the lesions and dynamically adjusted to a 3D volume of interest. The maximum standardized uptake value (SUV_max_) of FAPI was computed using the formula: maximum pixel value within the ROI activity (MBq kg^−1^) / (injected dose [MBq]/body weight [kg]).

### ^68^Ga‐FAPI‐04 PET/MR Imaging in Patients with AA

^68^Ga‐FAPI‐04 PET/MR examination involved the use of a uPMR790 scanner (United Imaging Healthcare Co. Ltd., Shanghai, China) with MRI sequences. Patients received an injection of ^68^Ga‐FAPI‐04 at a dose of 1.85 MBq kg^−1^. One hour post‐injection, a 15‐min PET scan for a one‐bed position was conducted in a 3D mode. Specialized multi‐sequence MR images (T1 and T2‐weighted images) were acquired. All PET data were reconstructed using time‐of‐flight information with 3D ordered‐subset expectation maximization protocol iterative reconstruction algorithms.

### ELISA

The Plasma FAP level of rabbits was measured using Rabbit FAP ELISA Kit (Kechengwei Biology, KCW‐060116R) according to the instructions. Plasma Inducible Nitric Oxide Synthase (iNOS) level was measured using Rabbit iNOS ELISA Kit (Hailing Biotechnology, HLE95105). Plasma interleukin‐1 beta (IL‐1β) level was measured using Rabbit IL‐1 beta ELISA Kit (Hailing Biotechnology, HLE95009) according to the instructions. The plasma FAP level of humans was measured using a human FAP ELISA Kit (Cloud‐Clone Corp, SEC469Hu). Similarly, the measurement of plasma iNOS level was conducted using a human iNOS ELISA Kit (CUSABIO, G09034678) following the provided protocol. Plasma IL‐1β level was measured using a human IL‐1 beta ELISA Kit (Servicebio, GEH0002) according to the prescribed guidelines.

### Western Blotting

All mice and New Zealand Rabbits samples were homogenized using RIPA Lysis Buffer (purchased from EpiZyme, PC101). Protein concentration was determined utilizing a BCA protein assay kit (Yeasen, 20201ES76). 10% acrylamide/bis‐acrylamide gels were used to detect levels of FAP, α‐SMA, and osteopontin (OPN). Protein extracts were boiled at 95 °C for 10 min in 5X loading buffer, subjected to SDS‐PAGE, and transferred to PVDF membranes (Meck Millipore, IPVH00010). After blocking the membrane with Protein Free Rapid Blocking Buffer (EpiZyme, PS108P) for 15 min at room temperature, the membranes were incubated with anti‐FAP (Abcam, ab314075; Thermo Fisher, BMS168), anti‐α‐SMA (ABclonal, A17910; Servicebio, GB12044), and anti‐OPN (Abcam, ab214050; ABclonal, A21084) antibodies at 4 °C overnight. The membranes were washed three times in (Tris‐buffered saline with 0.1% Tween 20) TBST buffer and incubated with goat anti‐rabbit IgG (Affinity, S0001) or goat anti‐mouse IgG (Affinity, S0002) secondary antibodies for 1 h at room temperature. The membranes were washed three times in TBST buffer and underwent chemiluminescent signals detection using Super ECL western blotting substrate (Yeasen, 36208ES60). Relevant outcomes were analyzed using ImageJ software and motivate protein levels were calculated by normalizing to the loading control GAPDH.

### Histological and Immunohistochemical Analysis

Human, rabbit, and mouse samples were fixed with 4% paraformaldehyde for 24 h and sectioned at 5 µm. Relevant sections were stained using Hematoxylin‐Eosin (H&E) Stain Kit (Sigma, 230251‐25G; BaSo, BA4041), Elastic Stain Kit (BaSo, BA‐4083B), and Masson Stain Kit (BaSo, BA‐4079B) according to manufacturer's instructions. Anti‐α‐SMA (ABclonal, A17910; Servicebio, GB12044) and anti‐OPN (Abcam, 214 050; ABclonal, A21084) were used to incubate the tissue sections and following the standard immunohistochemistry experimental steps. All images were quantified using ImageJ software.

### Immunofluorescent Staining

For translocator protein (TSPO) staining, a mitochondrial membrane protein that is implicated in the activated macrophages,^[^ ^27^ ^]^ was used to reflect the inflammation level of the aortic wall. The anti‐TSPO (Affinity, DF8227) was used to incubate the tissue sections and follow the standard immunofluorescent staining steps. Fluorescent co‐staining of FAP and α‐SMA was performed using the Tyramide signal amplification method following the relevant steps. All images were quantified using ImageJ software.

### Statistical Analysis

All experiment animals were assigned to groups randomly in a blinded fashion. For comparison between the two groups, unpaired two‐tailed Student's *t*‐test or Mann‐Whitney *U*‐test was used. For categorical variable analysis, a chi‐square test was used. For correlation analysis, a Pearson correlation test was used. A *p* value < 0.05 was considered statistically significant. All data represent mean ± SD. GraphPad Prism 9 and SPSS 26.0 were used for statistical analysis.

## Conflict of Interest

X.C. is a co‐founder of and holds shares in Yantal Lannacheng Biotechnology Co., Ltd.

## Supporting information



Supporting Information

## Data Availability

The data that support the findings of this study are available in the supplementary material of this article.
