# Supplementary material for: Fibroblast Activation Protein Acts as a Biomarker for Monitoring ECM Remodeling During Aortic Aneurysm via 68Ga‐FAPI‐04 PET Imaging
Source: Adv Sci (Weinh). 2025 Feb 14;12(14):2411152. doi: 10.1002/advs.202411152 (PMC11984865; doi:10.1002/advs.202411152)
Supplement: Supplementary file 1 — Supporting Information [file ADVS-12-2411152-s001.docx]

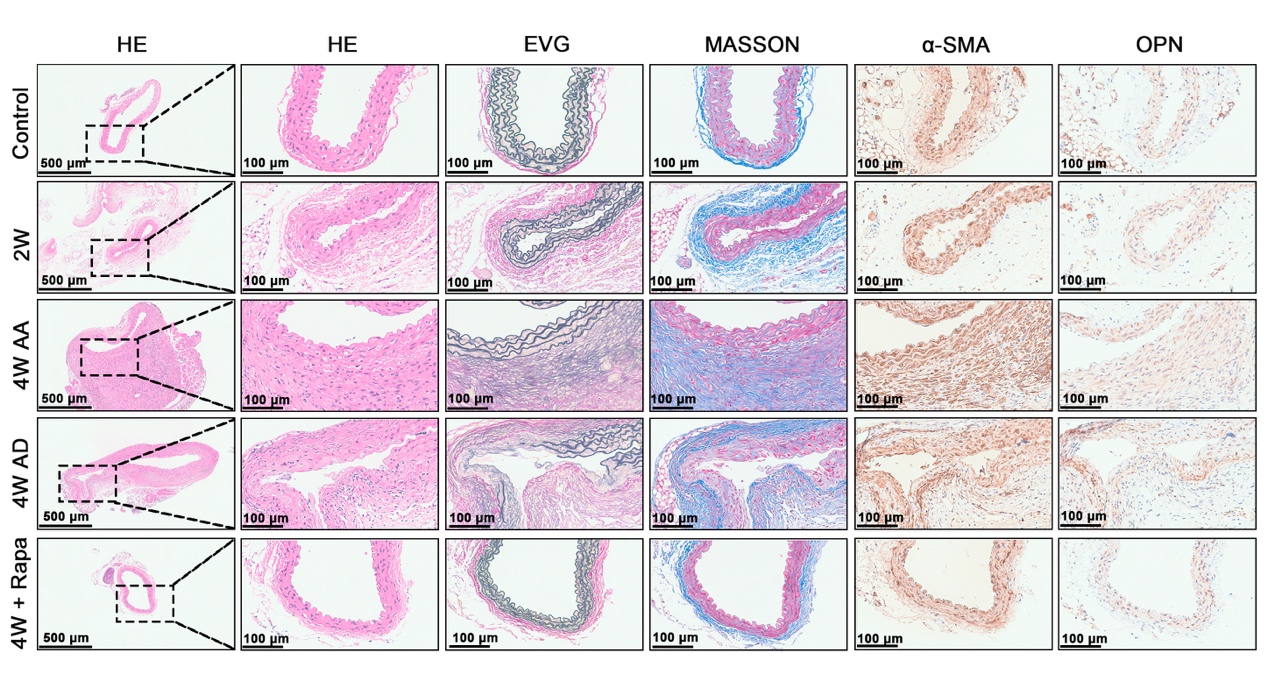


**Figure S1.** **Histology and immunohistochemistry of aortic specimens from different groups of *ApoE^-/-^* mice.** H&E, EVG and Masson staining and α-SMA and OPN immunohistochemistry of the indicated groups in the Ang II AA model. **AA**, aortic aneurysm; **AD**, aortic dissection; **α-SMA**, alpha smooth muscle Actin; **OPN**, Osteopontin; **EVG**, Verhoeff's Van Gieson; **Rapa**, rapamycin.


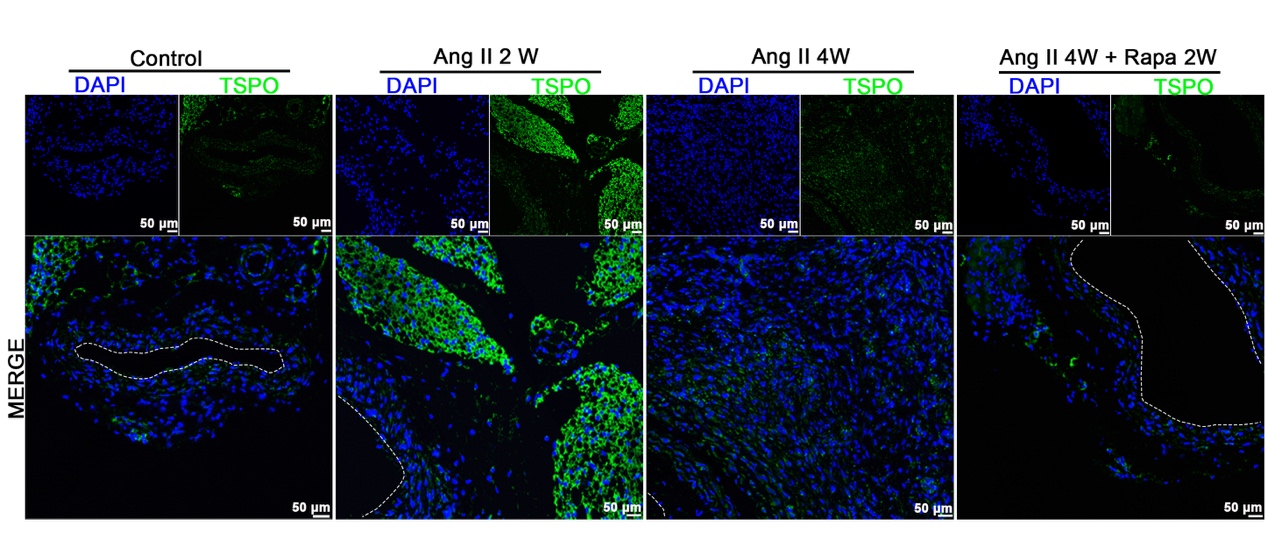


**Figure S2.** **Immunofluorescence analysis of TSPO from different groups of *ApoE*^-/-^ mice.** Immunofluorescence of TSPO in the Ang II AA mouse model of the indicated groups. **TSPO**, translocator protein; **Ang II**, Angiotensin II; **Rapa**, rapamycin.


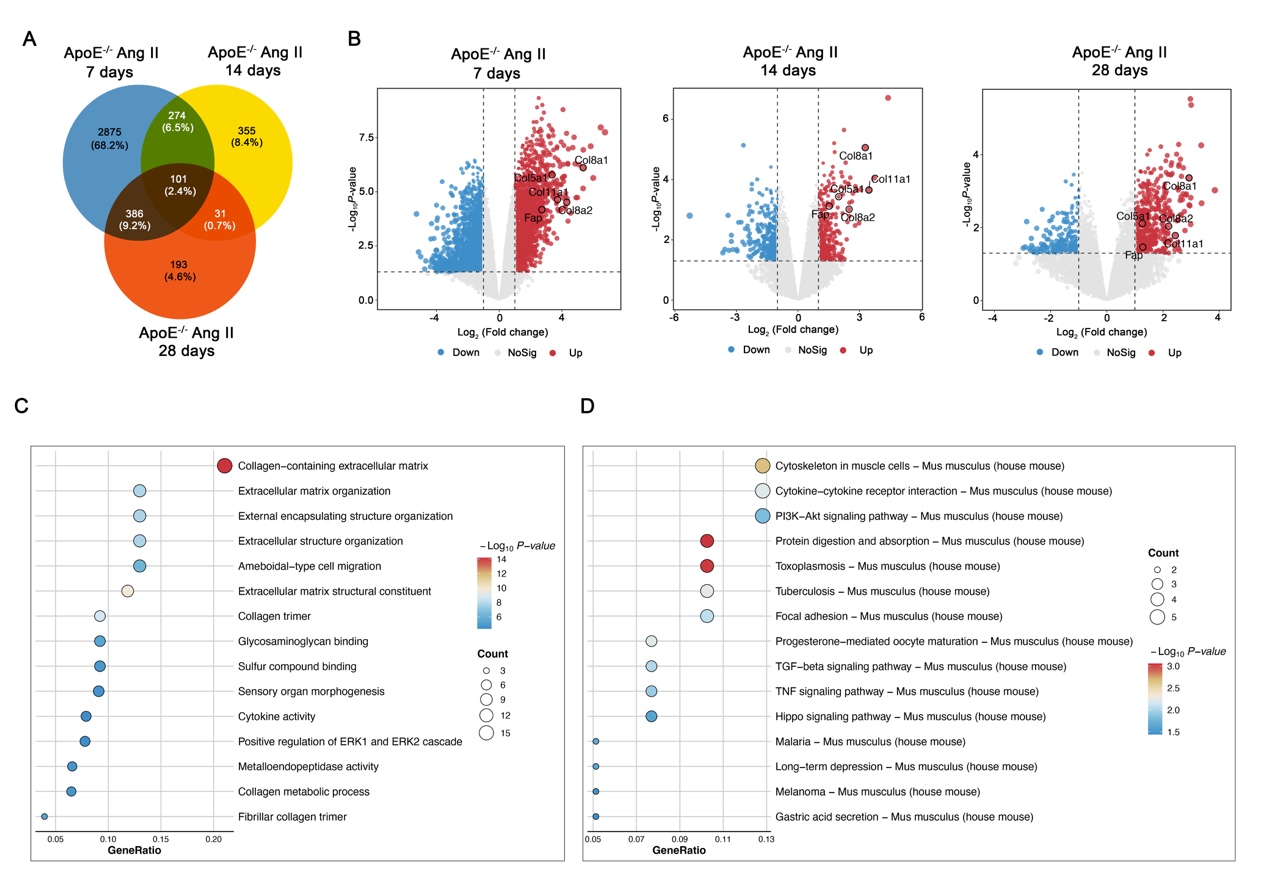


**Figure S3.** **The temporal trends of differentially expressed genes (DEGs) in *ApoE*^-/-^ AAA model.** (A) The intersection of the DEGs from suprarenal aorta of *ApoE*^–/–^ mice after ang II infusion for 7 days, 14 days, and 28 days. (B) Fap and related collagen genes such as Col5a1, Col8a1, Col8a2, and Col11a1, were consistent high expression within the aortic from 7 days to 14 days and 28 days. (C) GO analysis of the common DEGs from the three groups. (D) KEGG analysis of common DEGs from the three groups. **Ang II**, Angiotensin II; **GO**, Gene Ontology; **KEGG**, Kyoto Encyclopedia of Genes and Genomes.

**
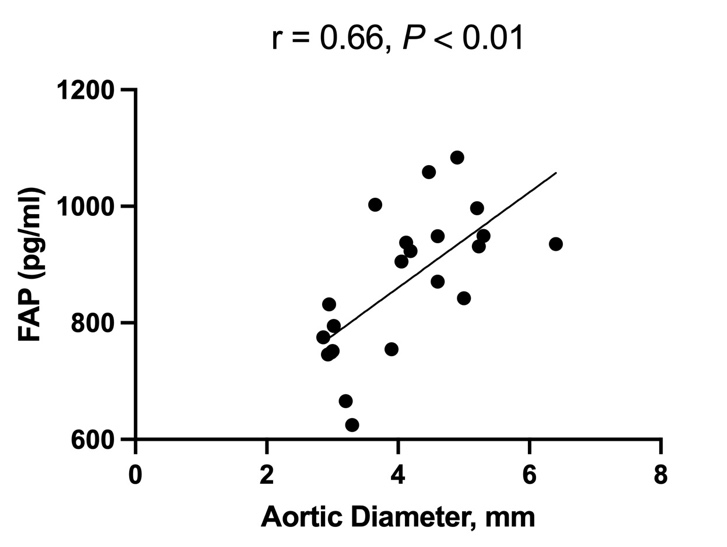
**

**Figure S4. The correlation between plasma FAP levels and aortic diameter in the rabbit AAA model.**


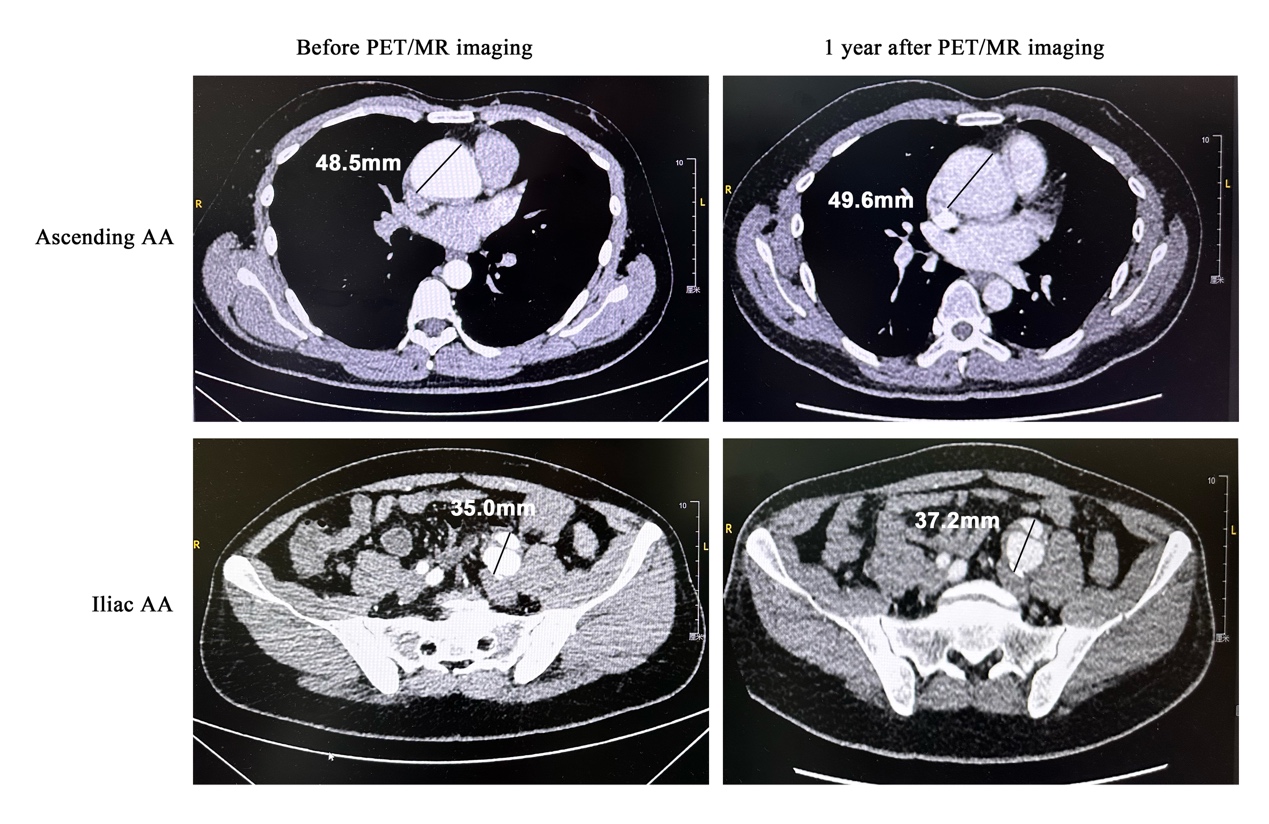


**Figure S5. The second case of CTA before ^68^Ga-FAPI-04 PET/MR imaging and one year after ^68^Ga-FAPI-04 PET/MR imaging.** The diameters of ascending AA and iliac AA from 48.5 mm to 49.6 mm and 35 mm to 37.2 mm, respectively. **CTA**, computed tomography angiography.

| Characteristics | Normal | Ascending AA | AAA |
| --- | --- | --- | --- |
|  | N=10 | N=10 | N=10 |
| Age, year | 60.7±10.0 | 60.3±10.1 | 41.7±12.2 |
| Sex, male | 7 | 8 | 7 |
| Hypertension, n | 2 | 2 | 4 |
| Diabetes, n | 0 | 2 | 4 |
| Smoking, n | 3 | 3 | 2 |
| Atherosclerosis, n | 0 | 2 | 4 |
| Tumor, n | 0 | 0 | 0 |
| Coronary Heart Disease, n | 0 | 0 | 0 |
| Connective tissue disease, n | 0 | 0 | 0 |

**Table S1. The Baseline characteristics of patients in the Figure 2C.**

| Baseline characteristics | First  case | Second  case | Third  case | Fourth  case | Fifth  case | Sixth  case |
| --- | --- | --- | --- | --- | --- | --- |
| Age, year | 73 | 41 | 29 | 57 | 69 | 61 |
| Sex, male | Male | Male | Female | Male | Male | Male |
| Hypertension | Yes | Yes | Yes | Yes | No | Yes |
| Smoking history | Yes | Yes | No | Yes | No | Yes |
| Aortic aneurysm type | | | | | | |
| Ascending AA  AAA  Iliac AA | - | + | - | - | + | + |
|  | + | - | + | + | - | - |
|  | - | + | - | - | - | - |
| Aortic aneurysm expansion rate (cm/yr) | | | | | | |
| Ascending AA  AAA  Iliac AA | - | 0.1 | - | - | 0 | 0 |
|  | 0.1 | - | unknown | 0.2 | - | - |
|  | - | 0.2 | - | - | - | - |
| TBR value of ^68^Ga-FAPI-04 | | | | | | |
| Ascending AA | - | 1.94 | - | - | 1.22 | 1.06 |
| AAA | 2.27 | - | 4.21 | 2.57 | - | - |
| Iliac AA | - | 2.6 | - | - | - | - |

**Table S2. The Baseline characteristics and TBR value of ^68^Ga-FAPI-04 in patients with AA.** The uptake levels of ^68^Ga-FAPI-04 were higher in patients who exhibited a higher expansion rate of AA. **AA**, aortic aneurysm; **AAA**, abdominal aortic aneurysm; TBR=Max(VOI_lesion_)/ Mean(VOI_Blood_).
